# Different Spectrophotometric Methods for Simultaneous Determination of Trelagliptin and Its Acid Degradation Product

**DOI:** 10.1155/2018/7370651

**Published:** 2018-01-30

**Authors:** Shereen Mowaka, Bassam M. Ayoub, Mostafa A. Hassan, Wafaa A. Zaghary

**Affiliations:** ^1^Analytical Chemistry Department, Faculty of Pharmacy, Helwan University, Ein Helwan, Cairo 11795, Egypt; ^2^Pharmaceutical Chemistry Department, Faculty of Pharmacy, The British University in Egypt, El-Shorouk City, Cairo 11837, Egypt; ^3^The Center for Drug Research and Development (CDRD), Faculty of Pharmacy, The British University in Egypt, El-Shorouk City, Cairo 11837, Egypt; ^4^Pharmaceutical Chemistry Department, Faculty of Pharmacy, Helwan University, Ein Helwan, Cairo 11795, Egypt

## Abstract

New spectrophotometric and chemometric methods were carried out for the simultaneous assay of trelagliptin (TRG) and its acid degradation product (TAD) and applied successfully as a stability indicating assay to recently approved Zafatek® tablets. TAD was monitored using TLC to ensure complete degradation. Furthermore, HPLC was used to confirm dealing with one major acid degradation product. The proposed methods were developed by manipulating zero-order, first-derivative, and ratio spectra of TRG and TAD using simultaneous equation, first-derivative, and mean-centering methods, respectively. Using Spectra Manager II and Minitab v.14 software, the absorbance at 274 nm–260.4 nm, amplitudes at 260.4 nm–274.0 nm, and mean-centered values at 287.6 nm–257.2 nm were measured against methanol as a blank for TRG and TAD, respectively. Linearity and the other validation parameters were acceptable at concentration ranges of 5–50 *μ*g/mL and 2.5–25 *μ*g/mL for TRG and TAD, respectively. Using one-way analysis of variance (ANOVA), the optimized methods were compared and proved to be accurate for the simultaneous assay of TRG and TAD.

## 1. Introduction

**Figure 1:**
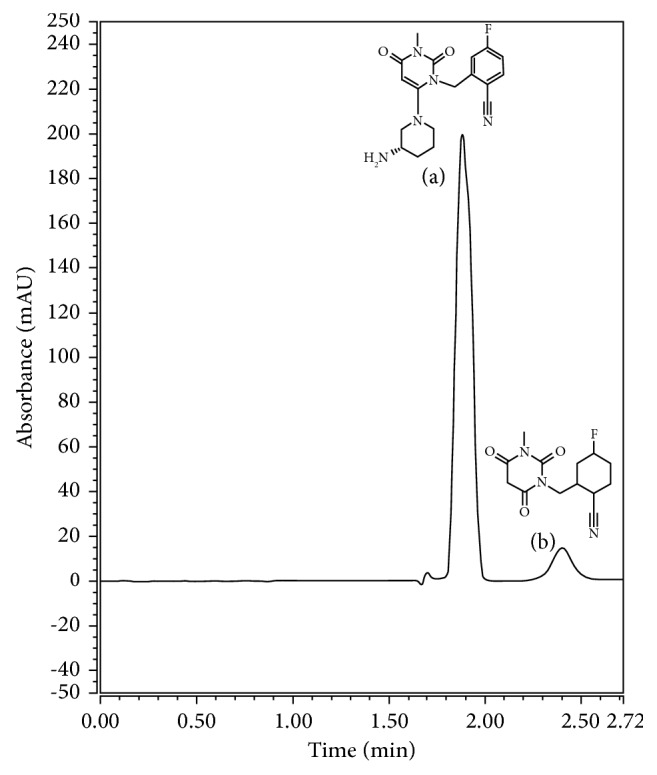
HPLC-UV chromatogram of laboratory-prepared mixture containing concentrations equivalent to 45 *µ*g/mL and 9 *µ*g/mL of TRG (a) and TAD (b), respectively.

Trelagliptin (TRG) (Figure 1) is a dipeptidyl peptidase-4 (DPP-4) inhibitor for treating type 2 diabetes mellitus as a once-weekly monotherapy. Its mechanism of action includes inhibiting the DPP-4 enzyme that leads to increment of glucagon-like peptide-1 (GLP-1) and other hormone levels [1]. TRG administration once weekly showed a high efficacy and good safety profile [2, 3]. TRG kinetic analysis revealed reversible and slow-binding inhibition of DPP-4, while X-ray diffraction data indicated a noncovalent interaction between DPP-4 and TRG [4, 5]. DPP-4 inhibitors are weight neutral and well tolerated and provide better glycaemic control for a longer period compared to conventional therapies. Despite the fact that various drugs are available, glycaemic control remains suboptimal in approximately half of patients with type 2 diabetes mellitus, one of the major reasons for low medication adherence [6–8]. A novel DPP-4 inhibitor, TRG, was approved in Japan in March 2015 and is the first once-weekly oral antidiabetic agent in the world. Medication adherence for the treatment of diabetes was reviewed in the recent phases I, II, and III clinical studies. TRG has demonstrated superiority to placebo and noninferiority to alogliptin, indicating its efficacy and tolerance in Japanese patients. TRG is expected to improve adherence and prevent complications. Due to the convenient dosing regimen, it is expected to be widely used in the clinical setting [6–9].

Although some LC methods were reported for quantification of TRG [10–12], no methods were found in the literature dealing with the direct UV assay of TRG in its pharmaceutical dosage form. In addition, no methods were used for the simultaneous assay of TRG and its acid degradation product (TAD) using UV spectrophotometric techniques. TAD (Figure 1) was found in the literature to be the main acid degradation product [12, 13], which is 2-[(3-methyl-2, 4, 6-oxo-tetrahydro-pyrimidin-1(2H) yl)-methyl]-4-fluorobenzonitrile. Literature results were ascertained by separating TRG from its acid degradation product on the TLC that results in two separated spots before the complete degradation and showed only one spot after finalizing the stress conditions as reported [12, 13]. Moreover, in this presented work, HPLC-UV was used to confirm that we have one major degradation product where chromatographic separation of TRG from TAD was obtained with retention times for TRG and its acid degradation product equal to 1.86 and 2.43, respectively (Figure 1).

The present work is considered as the first spectrophotometric methods for the analysis of TRG in Zafatek® tablets and for simultaneous determination of TRG in the presence of TAD that are considered as simple and inexpensive techniques. This study presents different methods resolving the overlapped spectra of TRG and TAD by manipulating their zero-order, first-order, and ratio spectra using simultaneous equation, first-derivative, and mean-centering methods, respectively.

## 2. Experimental

### 2.1. Instruments

UV spectrophotometer (S/N C367961148, Japan, JASCO) was used.

### 2.2. Reagents, Reference Samples, and Stock and Working Solutions

TRG was certified to contain more than 99.0%, and Zafatek tablets (each tablet contains 50 mg of TRG) were provided by Takeda Pharmaceuticals Ltd. (Japan). Stock solutions (1 mg/mL) and their appropriate dilution to working solutions (100 *µ*g/mL) of TRG were prepared separately in analytical grade methanol purchased from Sigma-Aldrich (Germany).

### 2.3. Sample Preparation

The coats of twenty Zafatek tablets were carefully removed; then, the tablets were powdered and mixed. An accurately weighed amount equivalent to 10 mg of TRG was transferred to a 100 mL volumetric flask, completed to volume with methanol, sonicated to dissolve, and filtered. Two, three, and four milliliters of the previously prepared extract were added separately to a series of 10 mL volumetric flasks and completed to volume with methanol. The final TRG concentrations were equivalent to 20, 30, and 40 *µ*g/mL.

### 2.4. Preparation of the Trelagliptin Acid Degradation Product (TAD)

Using TRG stock solution (1 mg/mL), acidic hydrolysis of TRG was carried out in a Fischer brand disposable tube by mixing 2.5 mL of TRG stock solution with 2.5 mL of 1 N HCl and was heated for 2 hours at 90°C. At the specified time (after cooling the tube contents), a precalculated amount of 1 N NaOH was added to neutralize the tube contents. Then, the tube contents were transferred into a 50 mL volumetric flask and completed to volume with methanol. Thus, the concentration of TAD was assumed to be 50 *μ*g/mL. Complete acidic hydrolysis of TRG was confirmed by injecting the sample into the HPLC-UV system that results in only one peak with a retention time of 2.43 (instead of TRG retention time that equals 1.86). Also, the sample was separated on TLC that results in only one spot that is corresponding to TAD. The sample was stored under 4°C until analysis.

### 2.5. Procedure

#### 2.5.1. Preliminary Investigation

**Figure 2:**
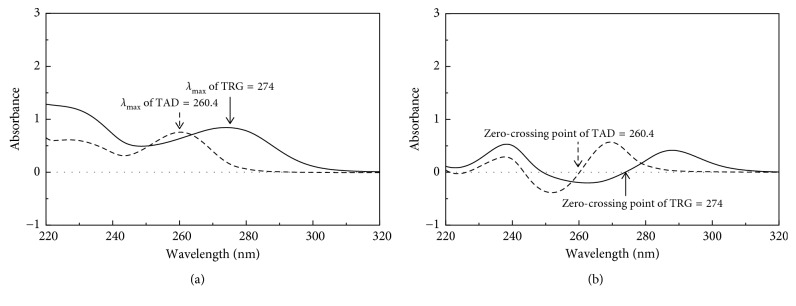
Overlay of the absorption spectra (a) and first-derivative spectra (b) of TRG 30 *µ*g/mL (solid line) and TAD 15 *µ*g/mL (dashed line), using methanol as a blank.

The zero-order absorption spectra of TRG (30 *µ*g/mL) and TAD (15 *µ*g/mL) were recorded separately against methanol as a blank. Overlay of both TRG and TAD spectra (Figure 2) showed the maximum absorption (*λ*_max_) at 274 nm and 260.4 nm, respectively. Overlay of the first-order spectra of TRG (30 *µ*g/mL) and TAD (15 *µ*g/mL) showed the TAD zero crossing point at 260.4 nm and the TRG zero crossing point at 274.0 nm, as shown in Figure 2.

#### 2.5.2. Linearity

Aliquots of working solutions corresponding to 50–500 *µ*g/mL and 25–250 *µ*g/mL of TRG and TAD, respectively, were added separately into a series of 10 mL volumetric flasks and completed to volume with methanol. The corresponding absorbance was measured at 274.0 nm and 260.4 nm for TRG and TAD, respectively, using methanol as a blank. Calibration curves (absorbance against concentration) were constructed for both TRG and TAD.

### 2.6. Simultaneous Equation Method

The simultaneous equation spectrophotometric method was successfully applied to TRG and TAD by manipulating their zero-order absorption spectra. TRG and TAD showed the maximum absorption (*λ*_max_) at 274 nm (*λ*_max_ 1) and 260.4 nm (*λ*_max_ 2), respectively. Absorbance at *λ*_max_ 1 and *λ*_max_ 2 was plotted against the corresponding concentrations of TRG and TAD, respectively, and calibration curves were constructed. Mixtures of TRG and TAD with different lambdas and concentrations of TRG and TAD (C_TRG_ and C_TAD_) can be calculated by a simultaneous equation. Through absorbance of the sample at *λ*1 and *λ*2 (*A*1 and *A*2), absorptivities of TRG at *λ*_max_ 1 and *λ*_max_ 2 (*a*_TRG_ 1 and *a*_TRG_ 2) and absorptivities of TAD at *λ*_max_ 1 and *λ*_max_ 2 (*a*_TAD_ 1 and *a*_TAD_ 2) are as follows:

(1) $$\begin{array}{c} C_{\text{TRG}}=\frac{\left[\left(A2*a_{\text{TAD}}\;\;1\right)-\left(A1*a_{\text{TAD}}\;\;2\right)\right]}{\left[\left(a_{\text{TRG}}\;\;2*a_{\text{TAD}}\;\;1\right)-\left(a_{\text{TRG}}\;\;1*a_{\text{TAD}}\;\;2\right)\right]},\\ C_{\text{TAD}}=\frac{\left[\left(A1*a_{\text{TRG}}\;\;2\right)-\left(A2*a_{\text{TRG}}\;\;1\right)\right]}{\left[\left(a_{\text{TRG}}\;\;2*a_{\text{TAD}}\;\;1\right)-\left(a_{\text{TRG}}\;\;1*a_{\text{TAD}}\;\;2\right)\right]}. \end{array}$$

The total absorbance is the sum of TRG and TAD absorbance, as their *λ*_max_ are relatively dissimilar and they do not interact chemically.

### 2.7. First-Derivative Method

The first-derivative spectrophotometric method was successfully applied to TRG and TAD by manipulating their first-order spectra. The amplitudes of the first-order spectra were measured for TRG and TAD at 260.4 nm and 274 nm, respectively. Then, the amplitudes were plotted against corresponding concentrations of TRG and TAD to construct calibration curves.

### 2.8. Mean-Centering Method

The scanned zero-order spectra of TRG and TAD were separately divided by 25 *µ*g/mL of TAD and 50 *µ*g/mL of TRG, respectively. Using the Minitab v.14 program, the obtained ratio spectra were mean centered and then, the mean-centered values of TRG and TAD were measured at 287.6 nm and 257.2 nm, respectively. Calibration curves were constructed by plotting the mean-centered values against corresponding concentrations.

#### 2.8.1. Accuracy and Precision

Three different ratios of the drug and its degradation product (TAD equals to 10%, 15%, and 20% of TRG) as 3 laboratory-prepared mixtures were applied using concentrations corresponding to 35, 40, and 45 *µ*g/mL and 3.5, 6, and 9 *µ*g/mL of TRG and TAD, respectively. The zero-order absorption spectra of the mixtures were recorded using methanol as a blank. Finally, the obtained spectra were manipulated by different methods to calculate the corresponding concentration of each drug. Furthermore, they were analyzed using the proposed methods three times within the same day (*n* = 3) and on three successive days (*n* = 3). The percent recoveries (% *R*) and the percent relative standard deviation (% RSD) were calculated for each method.

#### 2.8.2. Assay of Zafatek Tablets

The absorbance spectrum of the tablet extract prepared under Sample Preparation was recorded. Then, the percent recoveries and standard deviation were calculated.

## 3. Results and Discussion

**Figure 3:**
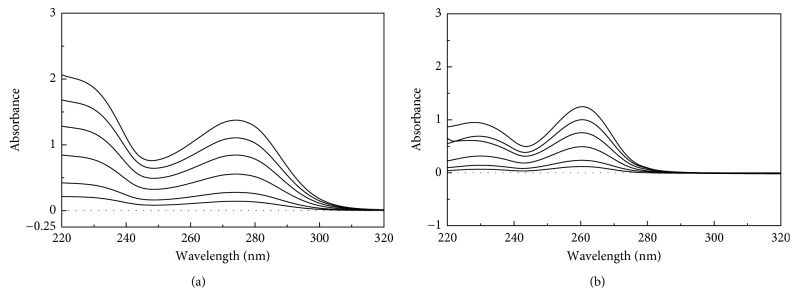
Overlay of the zero-order absorption spectra of TRG (5, 10, 20, 30, 40, and 50 *µ*g/mL) (a) and TAD (2.5, 5, 10, 15, 20, and 25 *µ*g/mL) (b), using methanol as a blank.

Calibration curves for the simultaneous equation method were constructed by plotting absorbance of TRG and TAD (Figures 3 and 3) against the corresponding concentrations. A simultaneous equation at the specified lambdas (274.0 nm and 260.4 nm) was used to get the corresponding concentration of TRG and TAD in their mixtures. Then, the percent recoveries of both TRG and TAD were calculated for the three laboratory-prepared mixtures (Table 1).

**Figure 4:**
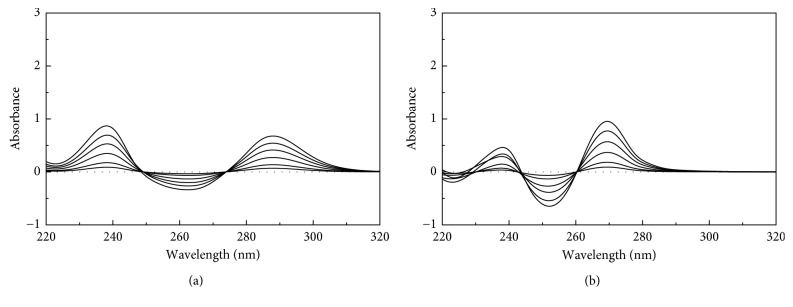
Overlay of the first-order spectra of TRG (5, 10, 20, 30, 40, and 50 *µ*g/mL) (a) and TAD (2.5, 5, 10, 15, 20, and 25 *µ*g/mL) (b), using methanol as a blank.

Calibration curves for the first-derivative method were constructed by plotting amplitudes of TRG and TAD (Figures 4 and 4) against the corresponding concentrations. The concentrations of TRG and TAD were calculated by applying the corresponding regression equations, as shown in Table 2.

**Figure 5:**
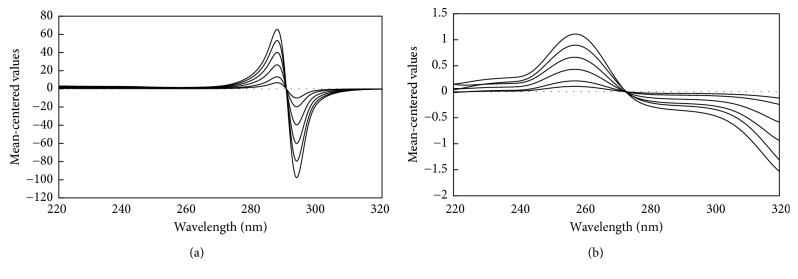
The mean-centered values of the ratio spectra representing 5–50 *μ*g/mL TRG divided by 25 *μ*g/mL of TAD (a) and the ratio spectra of 5–25 *μ*g/mL TAD divided by 50 *μ*g/mL of TRG (b), using methanol as a blank.

For the mean-centering method, the absorption spectra of TRG and TAD were separately divided by the zero-order spectra of 50 *μ*g/mL TRG and 25 *μ*g/mL TAD to get the ratio spectra that were mean centered using the Minitab program. Influences of different variables were studied, including divisor concentration and smoothing factor. Different concentrations were tried as divisors (30–60 *μ*g/mL of TRG and 15–35 *μ*g/mL of TAD). The selected divisors (50 *μ*g/mL TRG and 25 *μ*g/mL TAD) showed minimum noise, maximum sensitivity, and smoother ratio spectra, as shown in Figures 5 and 5. Mean centering was applicable, as TRG and TAD are noninteractive to each other, and each of them obeys Beer's law according to the following equation (*V*_*a*_ = A_TRG_ C_TRG_ + A_TAD_ C_TAD_), where *V*_*a*_ is the vector of absorbance, A_TRG_–A_TAD_ are the molar absorptivities, and C_TRG_–C_TAD_ are the concentrations of TRG and TAD, respectively. After division over A_TAD_, the resultant ratio spectra were mean centered; C_TAD_ will be zero value enabling the determination of C_TRG_ without interference from TAD and the same concept regarding A_TRG_, as shown in Table 3.

### 3.1. Validation according to ICH Guidelines

#### 3.1.1. Linearity

The linearity of the calibration curves was confirmed by LOD–LOQ parameters, STEYX, *S*_*b*_, and *S*_*a*_ as shown in Tables 1–3, where LOD is the limit of detection, LOQ is the limit of quantification, STEYX is the standard error of estimation, *S*_*b*_ is the standard deviation of the slope, and *S*_*a*_ is the standard deviation of the intercept. Also, the methods were adopted successfully for the assay of TRG and TAD in their mixtures. Acceptable results of the regression parameters were achieved as shown in Tables 1–3 [14].

#### 3.1.2. Accuracy and Precision

Accuracy was checked by calculating the percent recoveries of TRG and TAD in their laboratory-prepared mixtures, while precision values were checked using their intraday and interday records, *n* = 3, as shown in Tables 1–3.

#### 3.1.3. Specificity and Application on the Pharmaceutical Dosage Form

TRG was determined in its laboratory-prepared mixtures with TAD and in Zafatek tablets without interference from the excipients of the pharmaceutical dosage form. The mean of the percent recoveries and standard deviation were calculated, as shown in Tables 1–3.

### 3.2. Statistical Comparison

ANOVA comparison of the proposed methods at *p*=0.05 showed no significant difference, as shown in Table 4.

## 4. Conclusion

The optimized analytical methods were confirmed to be precise and accurate for determination of TRG and TAD based on the simple economic assay. The methods were applied successfully on the pharmaceutical dosage form, with acceptable validation results. Simultaneous determination for laboratory-prepared mixtures of TRG and TAD was achieved through manipulating their zero-order, first-order, and ratio spectra. The developed methods should be of interest to the analysts in the area of drug control and can be used by QC laboratories.

## Figures and Tables

**Table 1:** Results obtained by the proposed simultaneous equation spectrophotometric method for determination of TRG and TAD.

Item	TRG at 274 nm	TRG at 260.4 nm	TAD at 274 nm	TAD at 260.4 nm
Range of linearity (*μ*g/mL)	5–50	5–50	2.5–25	2.5–25
Regression equation	*A* = 0.0276 C·*μ*g/mL + 0.0056	*A* = 0.0208 C·*μ*g/mL + 0.0063	*A* = 0.0129 C·*μ*g/mL–0.0050	*A* = 0.0505 C·*μ*g/mL–0.0117
Regression coefficient (*r*)	0.9999	0.9997	0.9990	0.9999
LOD (*μ*g/mL)	1.01 *μ*g/mL	1.45	0.76	0.42
LOQ (*μ*g/mL)	3.05 *μ*g/mL	4.41	2.32	1.27
^∗^ *S*_*b*_	2.16 × 10^−4^	2.35 × 10^−4^	1.54 × 10^−4^	3.28 × 10^−4^
^∗^ *S*_*a*_	0.008	0.009	0.003	0.006
Confidence limit of the slope	0.0276 × 5.95 × 10^−6^	0.0208 × 4.89 × 10^−6^	0.0129 × 1.99 × 10^−6^	0.0505 × 1.66 × 10^−5^
Confidence limit of the intercept	0.0056 × 4.50 × 10^−5^	0.0063 × 5.46 × 10^−5^	−0.0050 × 1.42 × 10^−5^	−0.0117 × 7.11 × 10^−5^
Standard error of the estimation	0.009	0.008	0.003	0.006
*Accuracy* Laboratory prepared (mean ± SD)	100.66 ± 0.10		100.96 ± 0.86	
Standard addition (mean ± SD)	98.55 ± 1.70		—	
*Precision* Intraday % RSD	0.04–0.53–0.56		0.05–0.54–0.55	
Interday % RSD	0.40–0.54–0.92		0.45–0.52–0.92	
*Drug in dosage form* (mean ± SD)	100.98 ± 0.39		—	

^∗^Standard deviations of the slope *S_b_* & intercept *S_a_*.

**Table 2:** Results obtained by the proposed first-derivative spectrophotometric method for determination of TRG and TAD.

Item	TRG at 260.4 nm	TAD at 274 nm
Range of linearity (*μ*g/mL)	5–50	2.5–25
Regression equation	*A* = 0.0067 C·*μ*g/mL–0.0008	*A* = 0.0288 C·*μ*g/mL–0.0094
Regression coefficient (*r*)	0.9998	0.9997
LOD (*μ*g/mL)	1.21	0.77
LOQ (*μ*g/mL)	3.67	2.34
^∗^ *S*_*b*_	6.31 × 10^−5^	5.41 × 10^−4^
^∗^ *S*_*a*_	0.002	0.006
Confidence limit of the slope	0.0067 ± 4.23 × 10^−7^	0.0288 ± 9.93 × 10^−6^
Confidence limit of the intercept	−0.0008 ± 1.87 × 10^−6^	−0.0094 ± 6.05 × 10^−5^
Standard error of the estimation	0.002	0.006
*Accuracy* Laboratory prepared (mean ± SD)	99.43 ± 0.78	99.31 ± 0.45
Standard addition (mean ± SD)	99.51 ± 1.36	
*Precision* Intraday % RSD	0.45–0.53–0.71	0.43–0.51–0.64
Interday % RSD	0.28–0.41–0.51	0.27–0.39–0.46
*Drug in dosage form* (mean ± SD)	99.37 ± 0.89	

^∗^Standard deviations of the slope *S_b_* & intercept *S_a_*.

**Table 3:** Results obtained by the proposed mean-centering spectrophotometric method for determination of TRG and TAD.

Item	TRG at 287.6 nm	TAD at 257.2 nm
Range of linearity (*μ*g/mL)	5–50	2.5–25
Regression equation	*A* = 1.3134 C·*μ*g/mL + 0.2489	*A* = 0.0451 C·*μ*g/mL–0.0135
Regression coefficient (*r*)	0.9999	0.9999
LOD (*μ*g/mL)	0.98	0.42
LOQ (*μ*g/mL)	2.98	1.29
^∗^ *S*_*b*_	1.00 × 10^−2^	2.97 × 10^−4^
^∗^ *S*_*a*_	0.373	0.006
Confidence limit of the slope	1.3134 ± 1.32 × 10^−2^	0.0451 ± 1.34 × 10^−5^
Confidence limit of the intercept	0.2489 ± 9.29 × 10^−^	−0.0135 ± 7.45 × 10^−5^
Standard error of the estimation	0.392	0.006
*Accuracy* Laboratory prepared (mean ± SD)	99.99 ± 0.14	101.24 ± 0.08
Standard deviation (mean ± SD)	98.90 ± 1.75	
*Precision* Intraday % RSD	0.37–0.43–0.47	0.34–0.43–0.45
Interday % RSD	0.16–0.27–0.76	0.16–0.25–0.73
*Drug in dosage form* (mean ± SD)	98.92 ± 1.85	

^∗^Standard deviations of the slope *S_b_* & intercept *S_a_*.

**Table 4:** Statistical comparison between the proposed spectrophotometric methods.

Statistical term	TRG			TAD		
	Simultaneous equation	First derivative	Mean centering	Simultaneous equation	First derivative	Mean centering
Mean	100.66	99.43	99.99	100.96	99.31	101.24
SD±	0.10	0.78	0.14	0.86	0.45	0.08
% RSD	0.10	0.78	0.14	0.85	0.45	0.08
*n*	3	3	3	3	3	3
*V*	0.01	0.61	0.02	0.74	0.20	0.01

*Note*. The values in parentheses are the theoretical values at *p*=0.05. ANOVA results confirmed that there is no significant difference between groups of TRG (*F* = 5.350 and *p*=0.046) and TAD (*F* = 10.320 and *p*=0.011) at *p*=0.05.
